# Ethnic, racial and regional inequalities in access to COVID-19 vaccine, testing and hospitalization: Implications for eradication of the pandemic

**DOI:** 10.3389/fsoc.2022.809090

**Published:** 2022-08-09

**Authors:** Beth Maina Ahlberg, Hannah Bradby

**Affiliations:** ^1^Skaraborg Institute for Research and Development, Skövde, Sweden; ^2^Sociology Department, Uppsala University, Uppsala, Sweden

**Keywords:** vaccine hoarding, patent protection, neoliberalism, financialisation, inequalities, COVID-19

## Abstract

The COVID-19 pandemic has made visible inequalities as exemplified by unequal access to COVID-19 vaccine across and within countries; inequalities that are also apparent in rates of testing, disease, hospitalization and death from COVID-19 along class, ethnic and racial lines. For a global pandemic such as the COVID-19 to be effectively addressed, there is a need to reflect on the entrenched and structural inequalities within and between countries. While many countries in the global north have acquired more vaccines than they may need, in the global south many have very limited access. While countries in the global north had largely vaccinated their populations by 2022, those in the global south may not even complete vaccinating 70% of their population to enable them reach the so-called herd immunity by 2024. Even in the global north where vaccines are available, ethnic, racialized and poor working classes are disproportionately affected in terms of disproportionately low rates of infection and death. This paper explores the socio-economic and political structural factors that have created and maintain these disparities. In particular we sketch the role of neoliberal developments in deregulating and financializing the system, vaccine hoarding, patent protection and how this contributes to maintaining and widening disparities in access to COVID-19 vaccine and medication.

## Introduction

With more than 200 million known cases of COVID-19 and nearly 5 million deaths around the world as of September 2021 (Mancini and Burn-Murdoch, 2021; WHO, 2021), the COVID-19 pandemic presents a major global challenge. Nonetheless, while the pandemic no doubt presents a dark phase for humanity, there are also signs indicating some awakening. In the context of the lockdown implemented by governments in many countries and consequent hardships experienced by the people, mutual help groups for those indistress have emerged, especially among the youth (Wickramanayake, 2020). Bhattacharya (2020) notes how ordinary Italians sang to one another across balconies, expressing solidarity with neighbors living in isolation and caregivers on the frontlines. Moreover, at government level, welfare reforms appeared to be returning, leading some - for example, Monbiot (2020) - to predict the collapse of the neoliberal character of state-sponsored welfare. Neoliberalism or the economic ideology of capitalism, has, since the 1970s depleted public services, turning service institutions such as healthcare and education into commercial business, focused on profit accumulation for the few at the expense of poorly paid workers, ethnic and racialized groups and has thus aggravated inequalities between citizens and countries (Monbiot, 2017; Ahlberg et al., 2019).

### The rise of neoliberalism and the structuring of inequalities

Neoliberalism or the doctrine of the free market and related political and individual freedoms, was perhaps best articulated, by the economist Friedman (1962) who strongly opposed the type of liberal democracy that developed in the middle of the nineteenth century, with its emphasis on equality and social welfare, which he defined as state intervention and paternalism. Neoliberalism is a force explicitly aimed at the decay of the nation state and democratic welfare (Davidson and Saull, 2016). Moreover, it entails extending the doctrine of the free market to embrace every part of public and personal worlds and leads to the transformation of states and governments from being providers of social welfare to promoters of market and competition. Neoliberalism thus implies, as argued by Monbiot (2017), cutting expenditure on social services including education, healthcare, and other social infrastructure; reducing government regulation that can diminish private profits; selling state-owned enterprises, of common goods and replacing it with individual responsibility to work hard to succeed in becoming wealthy. This emphasis on individual responsibility thus creates a cloud to obscure the conditions of those who are for example, as noted by Njoku et al. (2021), already live in poor residential segregated areas with little hope of lifting themselves from poverty.

In the Washington Consensus of 1989, it was agreed by the International Monetary Fund (IMF), World Bank and USA Department of Treasury, that the neoliberal operations of the free market and a reduction of state involvement in welfare needed to be extended to countries in Africa and Latin America (Hurt, 2015). In other words, these international institutions promoted the adoption of market-led development strategies by countries of the global south, with the justification that resulting economic growth would then trickle-down to benefit all their people. The World Bank and the IMF promoted a neo-liberal economic development model forcing poor countries to institute structural adjustment programs (SAPS), that involved privatizing essential services (including healthcare), as a condition for receiving development aid, whether grants or loans. The assumption then was that leaving market mechanisms to their own devices would ensure competition, leading to economic growth (or at least poverty reduction), and by the 1980s these ideas had largely replaced the language of development. But, contrary to these expectations, the SAPs gave rise to economic policies that induced stagnation and deeper poverty. Bello and Ambrose (2006) argue that the conditionalities imposed by the IMF and the World Bank, that governments of poor countries cut spending on public institutions, cut subsidies to farmers, privatize public services such as health care, education, water and electricity, as a prerequisite for receiving “help” (including loans) is what deepened poverty. Nanda (2002) shows, for example, how in healthcare a user fee, introduced as part of cost recovery within a SAP led to decreased health service utilization in Ghana, Swaziland, Zaire and Uganda. In another twist, while the poor countries were forced to cut subsidies to their farmers, the rich countries in the north not only subsidize their farmers, but also in essence close their markets for products from the poor countries, while at the same time flooding the south with products that push local farmers out of business (Maren, 1997). This shift has been part of health and healthcare being regarded as a market commodity rather than a human right (Mayhew, 2002). Apart from poverty, another major challenge for African countries resulting from these different phases of modernization is the shaping of a leadership that has in turn, destroyed Africa through lack of foresight, mismanagement and corruption (Maathai, 2009), thereby contributing to what Olukoshi (2004) calls the erosion of the state. To have any hope of achieving the international goals around the right to health or addressing increased vulnerability, there is great need for critical reflection on what these neoliberal developments have entailed not only for the poor countries, but also for poor workers, ethnic and racialised groups.

Neoliberalism according to Davis (2013), thus results in a paradox where the poorest people have to find solutions for their collective health care, education, and social security and, should they fail, they are blamed as being lazy. It is this form of neoliberalism that Monbiot (2020) now argues is shifting with power migrating not just from private money to the state but also from the state to the people. But the triumph of the people is far from assured. Briggs et al. (2020) for example, describe current welfare interventions in the UK as the conservative government embracing socialism in order to save capitalism; a position also supported by Sumonja (2021) and Evans-Pritchard (2020). In their study, Briggs et al. (2020), describe how the lockdown suspended daily routines, with schools, pubs, cafes, restaurants and non-essential shops closed and people ordered to stay at home. As a result of the lockdown, it appeared that neoliberalism was being dispensed with, and significant state intervention in the economy was enacted to support businesses and workers. The government in the UK committed to paying wages (furlough), while mortgage freezes were arranged with banks, and self-employed workers received government assistance. These support measures seem to have lacerated the neoliberal ideology and Briggs and colleagues note what their study participants also reflected:

…*.COVID-19 represents an opportunity to evaluate our individual and collective priorities and envision an alternative future. Many people demonstrated “new hope” for change to what they saw as a politically impotent, unequal and ultimately flawed social system: their subjective dreams revolved around communal solidarity, a greener planet and a fairer society*.

In addition to the shift where communities in many parts of the world have mobilized, the lockdown or the new normal of working from home, schooling from home and reduced transport including air travel has, as argued by some, already lowered carbon emission and may improve health (Cicala et al., 2021) or at least has offered a glimpse of an alternative. Roy (2020), on the other hand argues that these shifts are not new because historically pandemics have always forced humans to break with the past and imagine their world anew. In this way, the COVID-19 pandemic is no different in the potential it offers as a gateway to a new world. While no doubt there are positive aspects of the COVID-19 pandemic, there are also challenges and, as Benach (2021. p. 51) argues:

…*. long-term confinement will have a negative impact on the mental and emotional health of the population, with the highly likely emergence of outbreaks of violence related to insecurity and social changes. One example is the case of women who must confine themselves together with their abusers. Another issue is that the virus is likely to remain with us, mutate, recur, or even become more virulent, and …., more severe pandemics may appear…*.

Whether the long term effects of the pandemic turn out to be progressive or regressive for humanity, there has been evidence of the short-term damage to particular socio-economic groups that has widened inequalities. The potential of what Marshall et al. (2021) call telework (working from home) after the lockdown did not apply to all. In the USA according to Marshall et al. (2021) there is a class difference:

*Households with members who teleworked more frequently reported higher levels of income and education and better health than those in which no one changed their typical in-person work in response to the COVID-19 pandemic*.

Moreover, home confinement has, according to OECD. (2021), also worsened population mental health markedly during the pandemic as the prevalence of anxiety and depression increased and even doubled in some countries as a result of isolation and unemployment. Furthermore, as elaborated by Allwood and Bell (2020), inequalities have widened in terms of who suffers from mental health problems. People already living with mental health problems and whose access to care has been interrupted by the pandemic, are at greater risk of worsening mental health.

Women and children who have been even more exposed to trauma and violence at home during the lockdown and people from ethnic groups where the prevalence of COVID-19 has been highest and the outcomes have been the worst have lost out due to the pandemic and the public health precautions that have been adopted. The disruption of employment and livelihoods has increased economic hardship most starkly amongst those with least to lose. These losses have been gendered, with women who have lost their jobs and earnings due to the pandemic becoming completely dependent on their partners, and girls who are stuck at home with no school, facing elevated levels of sexual and physical domestic violence with limited access to protection and treatment services as well as to justice for survivors. According to McCrary and Sanga (2021), domestic violence during the lockdown in USA increased 12% on average and 20% during working hours. Forced migrants to the global north are yet another group that, according to a study by WHO (2020), has low financial means, lacks access to healthcare due to uncertainty around entitlement and fear of deportation, such that care is not sought even in the case of suspected COVID-19 infection. The study reported significant negative impact of the pandemic on forced migrants' access to work, safety and financial means. The description of accumulating inequalities that have been apparent during the pandemic could continue: it is all too apparent that far from being a great leveler, the COVID-19 pandemic and response to it has entrenched rather than undone inequalities. It seems, as argued by Primrose et al. (2020), that political energies have been focused on managing the symptoms of COVID-19 rather than addressing the structural underpinnings of the inequalities that the pandemic highlighted. They note for example, that half of deaths worldwide have occurred in long term care homes, which operate commercially and include low-paid healthcare workers and personal caretakers. These were moreover least supplied with protective equipment and are also one category of workers who work even during lockdown and are therefore likely to be easily infected and also infect others.

During the shock of 2020, as the pandemic unfolded, hopes were then focussed on developing a vaccine and there was cause for optimism that the border-crossing nature of the viral transmission and the world-wide mortality would lead to meaningful global cooperation. Notwithstanding transmission ignoring national borders, vaccine production and distribution has shown stark inequality between nations. According to Gebrekidan and Apuzzo (2021):

*The rapid development of COVID-19 vaccines, achieved at record speed and financed by massive public funding in the United States, the European Union and Britain, represents a great triumph of the pandemic. Governments partnered with drug makers, pouring in billions of dollars to procure raw materials, finance clinical trials and retrofit factories. Billions more were committed to buy the finished product*.

In spite of being largely publicly funded, the COVID-19 vaccines are still privatized and monopolized, leaving pharmaceutical corporations the power to charge excessive prices for vaccines to maximize their profit (Marriot and Maitland, 2021). Furthermore, given the enormous investment by rich and powerful countries, it seems no wonder that vaccine hoarding may constitute a great and longstanding barrier to ever reducing global health inequalities. Where vaccines are available and have been taken up, COVID-19 mortality rates are reduced, implying that vaccines are effective. However, even in those countries where vaccines are available, not everybody has benefitted from them and, as argued by Njoku et al. (2021), racial and ethnic disparities in COVID-19 infection, hospitalization and mortality have not been undone by vaccination in USA. They note that:

*Black or African Americans, Hispanic or Latino persons, and American Indians or Alaska Natives….persons are more likely to become sick with, be hospitalized for, and die from COVID-19 when compared to non-Hispanic Whites*.

Two factors are also considered important in explaining these racial and ethnic disparities. The first is the residential segregation where black and other racial and ethnic minority groups are more likely to reside in neighborhoods with increased levels of poverty, less access to credit, employment, housing, transportation, educational and healthcare resources. This means they live in more health-limiting environments compared to Whites. Another factor in the USA in particular is the historical unethical procedures in research on Black people for example, the Tuskegee Syphilis study, which may have increased vaccine hesitancy among the Black people. Besides the racial and ethnic disparity in vaccine access within the rich countries, global disparities need to be addressed if the border-crossing of the virus is to be addressed. As Nyabola (2021) argues, the largest proportion of the global population is not vaccinated due to the effect of international politics, profiteering and domestic complacency. This suggests, as we have argued earlier, that there is need to critically reflect on the structural economic and political developments and their role in maintaining disparities. In the coming section we explore further the issue of vaccine hoarding and patent protection in extending and consolidating COVID-19 vaccine inequality.

### Vaccine hoarding and patent protection and global inequality in access to vaccine

According to WHO (2021), the rich countries with just 16% of the world's population have bought up to 60% of the world's COVID-19 vaccine supply, the aim being to vaccinate 70% of their adult population to secure herd immunity. By the end of June 2021, 46% of the people in high-income countries had received at least one COVID-19 vaccine while 20% in middle-income countries and only 0.9% of low-income countries were vaccinated (Rubin and Saidel, 2021). This discrepancy clearly indicates a global inequality in access to COVID-19 vaccines, which according to Ghosh (2021) and Gebrekidan and Apuzzo (2021), is due to a blatant vaccine grab by rich countries and the protection of patent rights by the same rich countries. Some rich countries in the global north have even ordered enough doses to vaccinate their populations ten times over. Canada, with a population of 38 million, has for example, reserved 414 million doses. Vaccine hoarding and, more so, patent protection have prevented wider production and therefore distribution of vaccines at prices that poor countries can afford. There are, according to Rubin and Saidel (2021), two schools of thought in the rich global north on patents. There are those who argue that patent protection is necessary in order to maintain incentives for pharmaceutical companies to innovate and invest in vaccine research and development. This school argues that without patent protection, the pharmaceutical companies would lose market to competitors and adversarial nations such as China. But according to Oxfam (2022), the incredible sums of money that governments have injected into the pharmaceutical corporations have driven asset prices up and with them created billionaire fortunes. Oxfam moreover notes how billionaires and corporations in food, energy, pharmaceuticals and technology sectors reap huge rewards while the cost of living is soaring and hurting many worldwide. Despite this, it is still argued that since poor countries lack infrastructure and expertise for effective domestic production, then they should take aid through voluntary commitments from industry, developed world governments and large NGOs. This, it is argued would be a more effective means to address the vaccine problem in the poor countries. The other school of thought advocates for waiving the patent and argues that removing patent protection is a necessity as companies located in high-income countries hold most, if not all, of the COVID-19 vaccines sold to governments, mostly in the rich global north. The price of these vaccines, combined with export restrictions and the inability of low and middle income, countries to manufacture their own vaccines at a lower price and without fear of litigation from patent holders, limits access to vaccines for the world's most vulnerable communities.

It is clear, as argued by Tran (2021), that the rich countries have mainly taken care of themselves first, without reflecting on the effects on global equality, let alone instituting effective pandemic precaution. Rich countries have started to issue booster shots, with about one million shots administered per day, which is three times the number of vaccines administered per day in low income countries (Mancini and Burn-Murdoch, 2021). The WHO has called for a moratorium on booster shots in the hopes of achieving 70% vaccination rates across all countries by the middle of 2022. Even countries such as Russia, China and India which have exported vaccines to other countries have done so as a way of building their own international clout rather than waiving the patents to allow production of vaccine by poor countries. This has become a point of competition as rich countries join the fray in shipping some of their hoarded vaccines to few poor countries.

There is thus need for a more expansive global vaccine manufacturing design if access to health is to realistically remain a human rights goal. In October 2020 India and South Africa led a group of low and middle income countries requesting the World Trade Organization (WTO) to waive certain Trade-Related Aspects of Intellectual Property Rights (TRIPS) provisions. However, the member states of the WTO failed to arrive at the required consensus to move forward with the proposed waiver, while the European Union, the United States, the United Kingdom, and other developed countries opposed the waiver request (Tran, 2021). According to Okoth (2022), a new draft agreement was circulated in early May 2022 after negotiations between the European Union (EU) and the United States for discussion at the WTO ministerial conference in Geneva on 12-15 June 2022. However, according to the civil Society organizations under the umbrella of the People's Vaccine Alliance, the process was flawed and untenable, because there was an apparent attempt by the EU to introduce amendments to the WTO text that critics saw as out of step with the original text proposed by India and South Africa at the beginning of the pandemic. South Africa and India, backed by 100 countries, had only called for a simple waiver on COVID-19 vaccine treatments and tests, which could have led to their manufacture in developing countries. There are also concerns that proposed new wording might prevent China, and perhaps Cuba that are capable of producing vaccines, from exporting to countries that need them.

Meanwhile, COVID-19 vaccines Global Access Facility COVAX, a vaccine-sharing scheme, was created to ensure that vaccines would reach all people everywhere. COVAX is led by the World Health Organization, Gavi (a public-private vaccine-promoting alliance) and the Coalition for Epidemic Preparedness Innovations (a foundation that finances research into vaccines for pandemics), and aims to ensure that all participating countries have access to inoculations. All countries in Africa have signed up to the scheme, which now has 190 members. Of these, 92 fall into the low- and middle-income group.

COVAX seeks to maximize the chances of successfully developing COVID-19 vaccines and manufacture them in the quantities needed to end the supply and distribution crisis. Thus, one motivation is humanitarian, but another is to prevent the emergence of new variants resistant to the available vaccines. According to COVAX, the target of distributing two billion doses by the end of 2021 will not be realized. Instead, COVAX expects to supply 1.4 billion doses of the vaccine in 2021, which is a shortfall of nearly a third (Diba et al., 2021). There are two main reasons for this failure. First, according to Horner (2021), some high-income countries in the global north, have started to roll out boosters as well as vaccinating children even before many low-income countries have distributed a first dose to all adults. Second, exports of COVID-19 vaccines from India which was the main supplier, were suspended and its output was redirected to domestic use to deal with a new devastating second wave of the virus in the country. The Serum Institute of India was due to supply COVAX with over a billion doses in 2021, but exports have still not resumed. Global vaccine inequality thus shows no sign of disappearing in the near future.

While the COVAX initiative did not get enough support from high income countries, billions of taxpayers' money from the same countries have been spent to help big pharmaceutical companies like AstraZeneca, Moderna and Pfizer BioNTech develop and produce vaccines. These as well as others, are the same companies that refuse to share their research, knowledge and technology with low income countries which means that other pharmaceutical companies, and especially those in low income countries with smaller budgets, cannot access the advances in science to step up their own vaccine production.

The issue of profit-making by private companies and the impact on healthcare and, ultimately inequalities in health outcomes, is demonstrated by the attempt to manufacture easy to handle and improved ventilators in the USA (Sanger et al., 2020). Although this was a case before the COVID-19 pandemic, it is relevant not just because the ventilator became central in the care of patients with COVID-19, but also because it demonstrates how powerful companies annihilate possible future competitors, and use public funding to support profit accumulation. In this case, the Department of Health and Human Services in USA, signed, according to Sanger et al. (2020), a contract in 2010 with a smaller company called Newport, based in California, but owned by a Japanese medical device company that only made ventilators. The agreement, with an initial payment of $6.1 million, was that the officials from the biomedical research agency would visit the firm making the ventilators every 3 months and the firm would submit monthly reports detailing its financial spending and progress. By April 2012, the Health and Human Service officials testified in Congress that the programme was on schedule for market approval by September 2013 after which, the ventilators would go into production. This however did not come to be, because the company that had signed the contract with the federal government was bought by a more powerful company called Covidien, which started asking for more funds from the government and an additional $1.4 million was granted. The government officials and executives from rival ventilator companies suspected that Covidien had acquired Newport to prevent it from making a cheaper product that would undermine profits that the larger company could make from selling its existing ventilators. In 2014, Covidien executives told the government officials that they would like to get out of the contract, complaining that it was not sufficiently profitable and the government agreed to cancel the contract. So, by the time of the arrival of COVID-19 at the beginning of 2020, ventilator manufacturing was unchanged compared with 2010.

It appears that the shortfalls noted above are a matter of the institutional power structures. Looking at the issues of vaccine nationalism and patent protection by companies in the rich global north, the problem lies in the political and economic structures and the ways the neoliberal capitalist system has been changing over time. As argued by Sell (2019) financialization and monopoly power are the main features of capitalism today. According to Goldstein (2009), financialization can be seen as a process that alters the fundamental aspects of capitalist micro and macro dynamics. Karwowski and Stockhammer (2017), add that financialization is closely linked to asset price, inflation and correlated with a debt-driven demand regime. The next section reflects on the impact of the changes in neoliberal capitalism as far as dealing with a global pandemic goes.

### Changing structural political economy and COVID-19 vaccine inequalities

As noted earlier, Monbiot (2020) argues that the neoliberalism model featuring deregulation, privatization, and the transformation of social protection regimes with faith in free markets is shifting. Accordingly, power is migrating not just from private money to the state but also from the state to the common people which may seem like a return to the Keynesian model of economic and social welfare. But there is need for more critical reflection on just how or in what ways neoliberal capitalism in the twenty-first century, has transformed to create the current vaccine inequalities seen within and between countries, where the rich countries are not just hoarding vaccines, but are also paying the big pharmaceutical companies for the vaccine development while failing to facilitate patent waivers, that would enable middle and low income countries to produce vaccines at lower costs. According to Sell (2019), the failure by rich countries in the global north to respond effectively to the COVID-19 pandemic has exposed the profound power of contemporary capitalism and thus offers an opportunity to rethink its role in shaping global health in the future. This is to say that for any change in the future, it is important to understand the structural features of the capitalist system that are not usually openly visible and ongoing transformations taking place in the capitalist system are crucial to grasp.

One aspect discussed by Pagano (2012) is the global monopolization of knowledge which creates hierarchical relations among firms and between capital and labor, since the capital-owners of some firms include exclusive ownership of much of the knowledge used in production. This is then supported by trade-related Intellectual Property Rights agreements. Intellectual monopoly capitalism is the dominant form of organization of big business, which as noted by Pagano (2012), has also transformed a world which has been mainly based on open science and open markets into a world of closed science. This transformation of the capitalist system has then closed markets and restricted the investment opportunities for many firms in different countries. Sell (2019) expands on the implications of the transformation of capitalism in the following way:

*Financialized capitalism is a pattern of accumulation in which profits accrue primarily through financial channels rather than through trade and commodity production. Financial markets, motives, institutions, and elites have increasingly come to dominate the global political economy, affecting everything from production and consumption, to regulation and health…*.

Some of the challenges arising from financialized capitalism include, according to Sell (2019), capital mobility which has facilitated tax evasion and the possibility of shifting revenue to tax havens or low-tax locations. As a result, this has reduced the tax base for funding programmes such as health care. The shift from commodity production and trade in goods to intangibles has, furthermore, meant that the major share of revenue goes to those who control the intangible assets such as financial products and intellectual property, all of which has also undermined the political power of labor and trade unions (Sell, 2019).

The capitalist transformation described above, informs the perspective presented by Kelly (2021) regarding how the big tech Companies such as Facebook, Netflix, Amazon, Apple, Google, Microsoft and others saw their stock prices soaring to record high during the nearly 2 years of the COVID-19 pandemic. Kelly (2021), describes this as the “black Swan event” where the pandemic pushed companies to send almost all of their white-collar professionals to work from home. Although an unintended benefit, working from home turned out to be a very successful consumption-expansion strategy for the companies mentioned above.

Another source of power for the giant tech companies is that they quietly buy up many companies, with most of the acquisitions going unreported and unannounced. This, as reported by De Vynck and Zakrzewski (2021), makes it harder to know how companies like Google and Apple shape the markets. A major question for law makers and government executives has been whether companies such as Amazon, Apple, Facebook, Google and Microsoft are too powerful to keep anti-competitive practices over markets. The law makers also worry over the rapid acquisition of other companies because of unforeseen effects on the economy. The US federal government requires that companies report acquisition of other companies worth more than $92 million, but from 2010 to 2019, the giants had together acquired 616 companies which were probably not reported: the giants are so rich they can afford to routinely buy start-up companies in order to obtain skilled employees, win innovative patents or simply eliminate potential competitors. This practice is similar to the case reported earlier, of buying up the small company called Newport which had signed a contract with the US government to produce cheaper and easy to use ventilators, in order to eliminate it. Apart from buying companies in part to stop competition, there is another aspect in what Fernandez and Klinge (2020) describe as corporate financialisation where for example, big pharmaceutical companies make little investment in productive capabilities or in research and development. Instead, they generate profits for shareholders at a scale that is socially unaffordable, and that precludes progressive change, such as supporting healthcare and patients around the world. Fernandez and Klinge further argue that the big pharmaceutical companies have increasingly become dependent on global market conditions of rising debt, dependence on mergers and acquisitions in order to replenish their drug patents. They therefore routinely block pro-health initiatives aimed at promoting the use of trade-related aspects of intellectual property rights' flexibilities to make essential medicines affordable to avoid threatening their profits and reducing shareholder value. Patent protection in turn increases the cost of drugs and reduces access to medicines and vaccines. This can be seen as what Marriot and Maitland (2021) describe as the great vaccine robbery. It is thus clear that major structural and policy reforms are needed to change this situation to enable all to have access to medicines.

## Discussion and conclusions

This paper is part of the research topic on the lessons learned from the COVID-19 pandemic so far that could help in building a fairer, healthier, inclusive and sustainable society. We have noted that there are positive trends as a result of the COVID-19 pandemic where communities in many areas have mobilized to support neighbors, healthcare workers and those who have been displaced. Governments have also taken action to support workers who lose their jobs and displaced communities in what Briggs et al. (2020) calls supporting socialism in order to protect capitalism. Even Monbiot (2020), who notes the shifting power of neoliberalism and community resurgence for collective support, also notes that there is no guarantee that the resurgence of collective action will survive after the pandemic and that the state is needed for providing health, education and an economic safety net, to distribute wealth and prevent private interests becoming too powerful. Sumonja (2021) notes that some neoliberal states around the world are using the fight against COVID-19 pandemic to improve the conditions of the working class.

However, although states may use the pandemic to improve some conditions for the working class, the quest for profitability forces firms to continuously reduce their labor costs, which as Sell (2019) argues, has increased income inequalities. Given the way the financialised capitalism has aggravated income inequalities, the need for states to introduce regulations to change this is of great importance. Some suggest that a way out of this is to introduce regulation in the banking sector including reducing the size of the “too big to fail” banks, imposing taxes on financial transactions to increase public revenue. For pharmaceutical companies, Sell (2019) states the need to curb the abuses of monopoly power through pricing transparency and price reduction for medicines to help increase access to essential medicines. Perhaps the major lesson is what Sumonja (2021) calls emergency Keynesianism to which governments around the world have resorted. However, given that the same governments in the rich global north also pay the pharmaceutical companies to develop vaccines which these countries then hoard, the death of neoliberalism in the near future seems unlikely.

While studying the financialized interests of capital does not necessarily hold interest for medical sociologists, the evidence of this pandemic suggests that we cannot afford not to pay attention.

## Author contributions

Both authors have contributed to the work and approved it for publication.

## Conflict of interest

The authors declare that the research was conducted in the absence of any commercial or financial relationships that could be construed as a potential conflict of interest.

## Publisher's note

All claims expressed in this article are solely those of the authors and do not necessarily represent those of their affiliated organizations, or those of the publisher, the editors and the reviewers. Any product that may be evaluated in this article, or claim that may be made by its manufacturer, is not guaranteed or endorsed by the publisher.
